# Reprogramming and multi-lineage transdifferentiation attenuate the tumorigenicity of colorectal cancer cells

**DOI:** 10.1016/j.jbc.2023.105534

**Published:** 2023-12-10

**Authors:** Tongtong Guo, Juan Wang, Maogui Pang, Wanning Liu, Xiaohui Zhang, Ahui Fan, Hengtao Liu, Qianqian Liu, Tianying Wei, Cunxi Li, Xiaodi Zhao, Yuanyuan Lu

**Affiliations:** 1 State Key Laboratory of Cancer Biology and National Clinical Research Center for Digestive Diseases, Xijing Hospital of Digestive Diseases, Fourth Military Medical University, Xi'an, Shaanxi, China; 2 Department of Gastroenterology, Tangdu Hospital, Fourth Military Medical University, Xi'an, Shaanxi, China; 3 Jiaen Genetics Laboratory, Beijing Jiaen Hospital, Beijing, China; 4 Cytogenetics Laboratory, Beijing Institute of Human Genetics and Reproduction Medicine, Beijing, China

**Keywords:** CRC, iPSC, transdifferentiation, tumorigenicity, therapy

## Abstract

Significant advances have been made in reprogramming various somatic cells into induced pluripotent stem cells (iPSCs) and in multi-lineage differentiation (transdifferentiation) into different tissues. These manipulable transdifferentiating techniques may be applied in cancer therapy. Limited works have been reported that cancer cell malignancy can be switched to benign phenotypes through reprogramming techniques. Here, we reported that two colorectal cancer (CRC) cell lines (DLD1, HT29) could be reprogrammed into iPSCs (D-iPSCs, H-iPSCs). D- and H-iPSCs showed reduced tumorigenesis. Furthermore, we successfully induced D- and H-iPSCs differentiation into terminally differentiated cell types such as cardiomyocyte, neuron, and adipocyte-like cells. Impressively, the differentiated cells exhibited further attenuated tumorigenesis *in vitro* and *in vivo*. RNA-Seq further indicated that epigenetic changes occurred after reprogramming and transdifferentiation that caused reduced tumorigenicity. Overall, our study indicated that CRC cells can be reprogrammed and further differentiated into terminally differentiated lineages with attenuation of their malignancy *in vitro* and *in vivo*. The current work sheds light on a potential multi-lineage differentiation therapeutic strategy for colorectal cancer.

Colorectal cancer (CRC), one of the most frequently occurring cancers in the world, ranks second in terms of global cancer mortality rate (1). In particular, *KRAS or BRAF* is frequently mutated in metastatic CRC, which confers a poor prognosis to patients with CRC (2). Significant progress in the treatment of CRC has been achieved through the use of monoclonal antibodies and small-molecule inhibitors, but their effects have never lasted very long (3, 4). Therefore, new treatments for CRC still need to be developed.

Cancer cells exhibit epigenetic plasticity, which indicates that cancer cells may recover the function of benign cells through the re-expression of certain genes. Expression of the Yamanaka transcription factors in tumor cells will change the epigenetic state of the cells, which leads to reprogramming, reprogramming into iPSCs, and reduction or even loss of malignancy (5, 6, 7, 8). For example, patient-derived melanoma cells and murine-derived melanoma cells have been reprogrammed to iPSCs with a benign phenotype, and reprogramming is more efficient than that of mouse fibroblasts (9, 10). Therefore, the reprogramming of cancer cells may be a promising strategy for cancer treatment that induces a transition from malignant to benign.

In a study conducted in 2017, researchers successfully established iPSC models of acute myeloid leukemia (AML) that retained their original genetic characteristics, regained the ability to cause leukemia *in vivo* after differentiation into hematopoietic cells, and reconstructed leukemia DNA methylation and gene expression patterns. However, when they differentiate into nonhematopoietic lineages, such as neurons and cardiomyocytes, the cells are no longer tumorigenic (11). Hepatocyte nuclear factor 1A (HNF1A), HNF4A, and forkhead box A3 (FOXA3) can reprogram human fibroblasts into liver-like cells (12, 13). This combination of three molecules may also reprogram hepatocellular carcinoma (HCC) cells into liver-like cells with precancerous functions. Due to reprogramming, the function of hepatocytes is gradually enhanced, and tumorigenic properties are gradually lost *in vitro* (14). All these findings suggest that reprogramming may be a potential breakthrough in cancer treatment.

Inspired by these studies, we wondered whether a similar approach could be applied to convert CRC cells into normal cells or cells with reduced malignancy. We introduced the combination of the reprogramming factors OCT3/4, SOX2, GLIS1, KLF4, and C-MYC into the human CRC cell lines DLD1, which carries *KRAS* mutations, and HT29, which carries *BRAF* mutations. These cells with *KRAS* or *BRAF* mutations could be successfully reprogrammed and showed typical characteristics of pluripotent stem cells. Furthermore, the reprogrammed iPSCs were successfully differentiated *in vitro* into terminally differentiated lineages, including neuron, cardiomyoid, and adipocyte-like cell types. The cardiomyocytes lost their tumorigenicity *in vivo*.

## Results

### Successful reprogramming of CRC cell lines into iPSCs using ReproRNA-OKSGM

**Figure 1:**
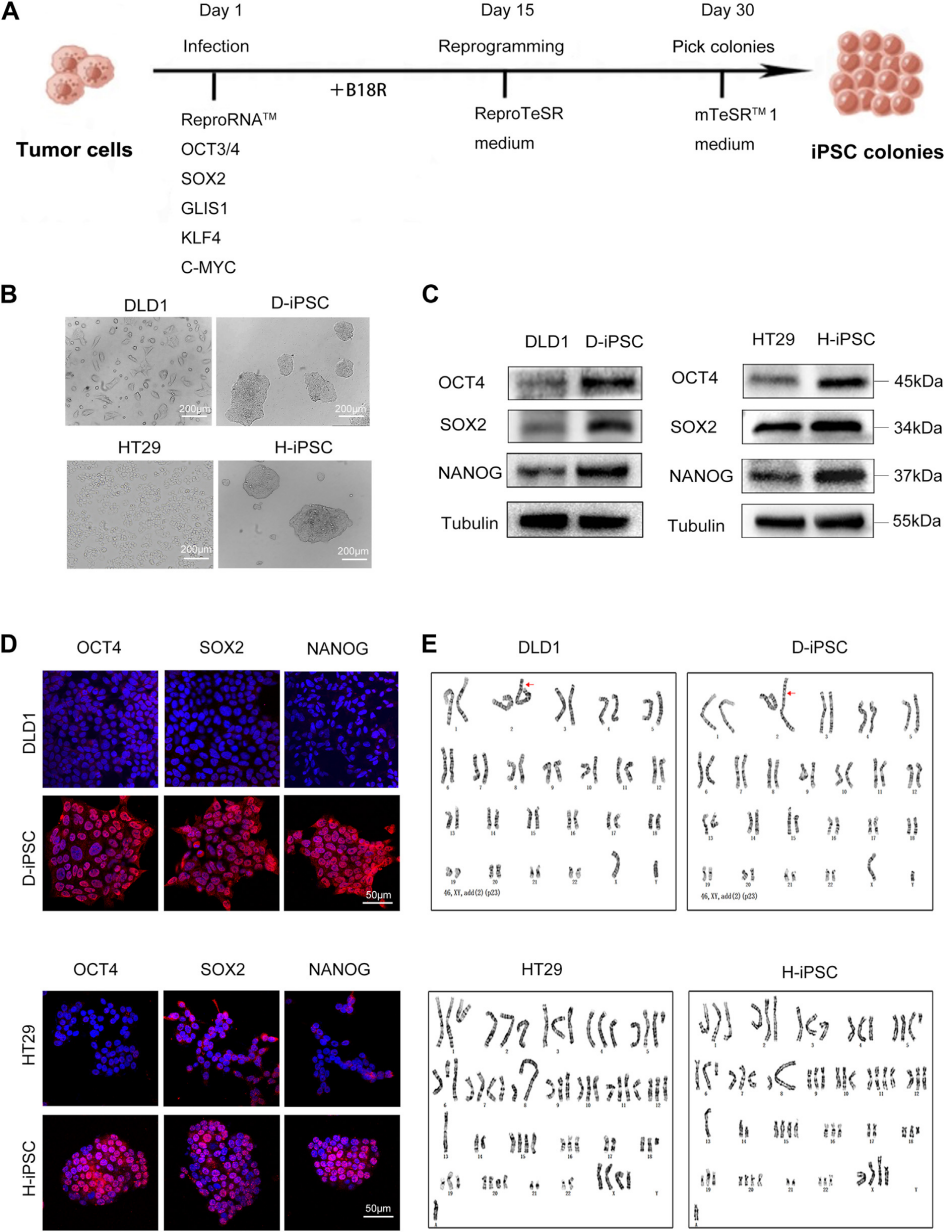
**Generation and validation of CRC-iPSCs**. *A*, reprogramming steps of colorectal cancer cells. *B*, the reprogrammed colorectal cancer cells showed ESC-like morphological features: the cells grew as colonies with smooth edges and dense centers. Scale bars, 200 μm. *C*, the Western blot results showed higher expression of the pluripotency markers OCT4, SOX2, and NANOG in CRC-iPSCs. Tubulin was used as an endogenous control. Blots are representative of three independent experiments. *D*, immunofluorescence staining of OCT4, SOX2, and NANOG in parental CRC cells and CRC-iPSCs. Nuclear staining was performed using DAPI. Scale bars, 50 μm. Images are representative of three independent experiments. *E*, A karyotypic analysis demonstrated the chromosome sets of CRC-iPSCs in parallel with their parental cells.

Takahashi and Yamanaka first reprogrammed human skin fibroblasts using retroviral vectors expressing the transcription factors OCT3/4, SOX2, KLF4, and c-MYC (15). Since then, multiple vectors expressing various types of transcription factors that can reprogram various cells have emerged rapidly with the aim of identifying more efficient and safer transfection methods (16, 17, 18, 19, 20). However, vectors are usually scavenged during host cell division, which may significantly reduce the pluripotency of iPSCs (21, 22), or the persistence of vectors limits their potential applications for laboratory safety requirements (23, 24). Thus, Yoshioka *et al*. developed a self-replicative RNA (srRNA) through the addition of B18R during skin fibroblast reprogramming (25, 26). Here, we used srRNA as a vector containing ReproRNA-OKSGM to reprogram the CRC cell lines DLD1 and HT29 (Fig. 1*A*). The reprogrammed cells developed into ESC-like colonies within approximately 30 days and were transferred to the Matrigel-coated matrix for passage. These iPSC-like cells showed the morphological characteristics of ESCs with colony growth and distinct boundaries (Fig. 1*B*).

We named these iPSC-like cells reprogrammed from DLD1 and HT29 cells D-iPSCs and H-iPSCs, respectively. Western blot analysis showed that stem cell-related markers, including OCT4, SOX2, and NANOG, were highly expressed in D-iPSCs and H-iPSCs (Fig. 1*C*). Additionally, the immunofluorescence assay detected strong signals of OCT4, SOX2, and NANOG in the nuclei (Fig. 1*D*), indicating the successful establishment of D-iPSCs and H-iPSCs. Chromosomal abnormalities occur frequently during the iPSC culture process, so we conducted a chromosomal karyotype analysis on D-iPSCs and H-iPSCs in parallel with their parental cells and found no additional chromosome abnormalities (Fig. 1*E*). These data suggest that CRC cells were successfully reprogrammed into iPSCs while maintaining the original genetic background.

### D-iPSCs and H-iPSCs are less malignant than their parental cell lines

**Figure 2:**
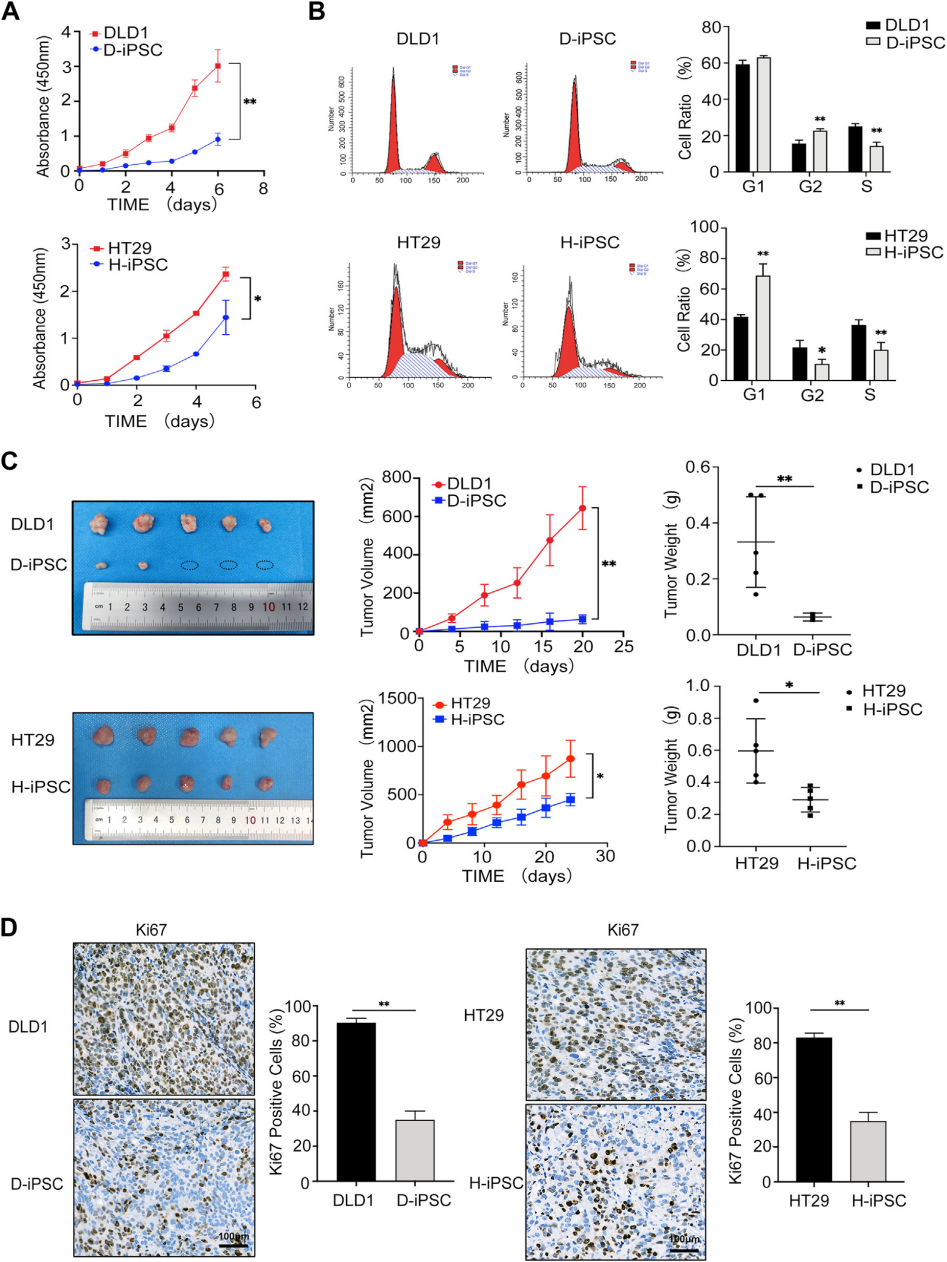
**Malignant phenotypic characteristics of CRC-iPSCs**. *A*, a CCK-8 assay was performed to detect the proliferation ability of CRC-iPSCs and parental CRC cells; n  =  5 for each group. (∗*p* < 0.05, ∗∗*p* < 0.01). *B*, flow cytometry was performed to assess the cell cycle, and a representative image is shown (*left*). The cell ratio was calculated for each period (*right*). (∗*p* < 0.05, ∗∗*p* < 0.01). *C*, the two pictures on the left show that the xenograft tumors were derived by injecting CRC-iPSCs and CRC cells; the volume changes of the xenograft tumors measured every 3 days were diagrammed as a tumor growth curve (*middle*), and the tumor weights are shown on the right. (∗*p* < 0.05, ∗∗*p* < 0.01). *D*, immunohistochemical analysis of the expression of the proliferation marker Ki67 in DLD1 cells, HT29 cells, and D-iPSCs, H-iPSCs. Scale bars, 100 μm.

Considering the malignant phenotype of induced iPSCs, we first assessed the proliferation capability of DLD1 cells, HT29 cells, D-iPSCs, and H-iPSCs using the CCK-8 assay. The D-iPSCs and H-iPSCs had a significantly lower proliferation capacity than the parental control cells (Fig. 2*A*). The flow cytometry data also showed that more cells within the D-iPSC and H-iPSC populations were arrested in the G1 phase (Fig. 2*B*). These data suggested that DLD1 and HT29 cells showed a weakening of malignant features *in vitro* after reprogramming. To further verify this finding *in vivo*, 5 × 10^6^ DLD1 cells and D-iPSCs or HT29 cells and H-iPSCs were injected subcutaneously into the dorsal flanks of nude mice (n = 5). We found that DLD1 and HT29 cells formed subcutaneous tumors as early as the 10th day after injection. We did not observe the appearance of dorsal tumors in nude mice injected with D-iPSCs and H-iPSCs until 2 weeks. The tumorigenicity of the reprogrammed cells was markedly reduced as judged by their tumor size, volume, and weight (Fig. 2*C*). Ki-67 immunohistochemical staining of the xenotransplantation of CRC cells and iPSCs is shown in Figure 2*D*, and the experimental results showed that the proliferation ability of CRC cells was significantly reduced after reprogramming. Thus, the proliferation ability of CRC cells after reprogramming is greatly reduced *in vivo* and *in vitro*.

### Successful differentiation of iPSCs into neuron-like cells

**Figure 3:**
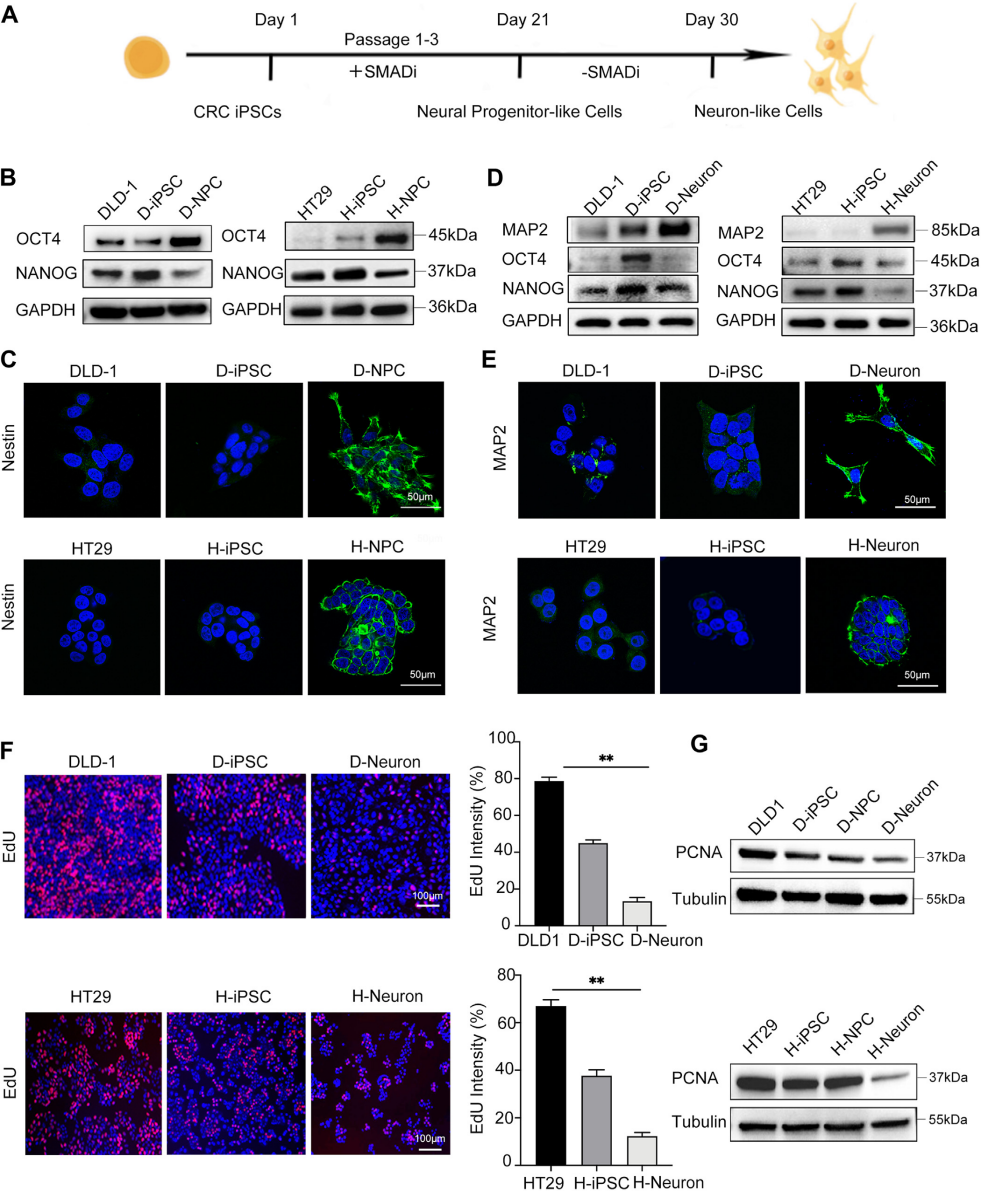
**CRC-iPSCs can effectively differentiate into neuron-like cells.***A*, scheme of the differentiation of CRC-iPSCs into neuron-like cells. *B*, the Western blot results demonstrated the expression of the stem cell-related markers OCT4 and NANOG. GAPDH was used as an endogenous control, and DLD1 and HT29 primary cells were used as negative controls. Blots are representative of three independent experiments. *C*, immunofluorescence showed that the induced neural cells expressed the neural progenitor cell marker Nestin. Scale bars, 50 μm. Images are representative of three independent experiments. *D*, the Western blot results demonstrated that the expression of the stem cell-related markers OCT4 and NANOG was decreased in neuron-like cells. Neuronal markers (MAP2) were highly expressed in induced neuron-like cells. Tubulin was used as an endogenous control, and parental DLD1 and HT29 cells were used as a negative control. Blots are representative of three independent experiments. *E*, immunofluorescence showed that the induced neuron-like cells expressed the neuronal marker MAP2. Scale bars, 50 μm. Images are representative of three independent experiments. *F*, for the labeling of proliferating cells, control, and neuron-like cells were incubated with EdU for 72 h. Representative image panels (*left*) and integrated intensity percentile charts (*right*) of EdU (*red*) and DAPI (*blue*) staining quantified using ImageJ are displayed, Mean ± SEM (n = 3); (∗*p* < 0.05, ∗∗*p* < 0.01). Scale bars, 100 μm. Images are representative of three independent experiments. *G*, the Western blot results showed that PCNA was expressed at low levels in neuron-like cells. Tubulin was used as an endogenous control. Blots are representative of three independent experiments.

We evaluated whether these established iPSCs could eventually differentiate into other cell lineages *in vitro*. We applied the method of inducing iPSCs to differentiate into neurons based on dual SMADi inhibition (27), which was a two-step differentiation process (Fig. 3*A*). In this process, we kept parental colorectal cancer cell lines and induced iPSCs in the same differentiation medium.

First, D-iPSCs and H-iPSCs were differentiated into neural progenitor cells. Within the first 21 days, the pluripotent marker OCT4 was upregulated, but these cells did not express the pluripotency marker NANOG (Fig. 3*B*). The neural progenitor cell marker, neuroepithelial stem cell protein (Nestin), was detected by immunofluorescence, and the results showed that the differentiated neural progenitor cells expressed higher levels of Nestin than the parental control cells (Fig. 3*C*). Thus, we successfully obtained neural progenitor cells, which could be induced into neurons. The neural progenitor cells differentiated from D-iPSCs and H-iPSCs were named D-NPCs and H-NPCs, respectively.

We then attempted to differentiate D-NPCs and H-NPCs into neurons, which were named D- and H-neurons. Western blot analysis showed that neurons, as terminally differentiated cells, showed lower expression levels of the stem cell-related markers OCT4 and NANOG than iPSCs and their parental cells (Fig. 3*D*). After 30 to 50 days, the expression of microtubule-associated protein 2 (MAP2) was also successfully detected by immunofluorescence, indicating neuron-like differentiation (Fig. 3*E*).

In addition, we detected the slow proliferation of neuron-like cells. As observed in Figure 3*F*, the 5-ethylene-2′-deoxyuridine (EdU) incorporation test showed a marked loss of proliferation ability after differentiation into neuron-like cells. PCNA was expressed lower in iPSCs and terminally differentiated neurons than in their parental cells (Fig. 3*G*), which suggested an inhibited proliferation status among iPSCs and differentiated neuron cells. All the above results indicate that the proliferation ability of D-neurons and H-neurons is further weakened compared with that of iPSCs.

### Successful differentiation of iPSCs into adipocyte-like cells

**Figure 4:**
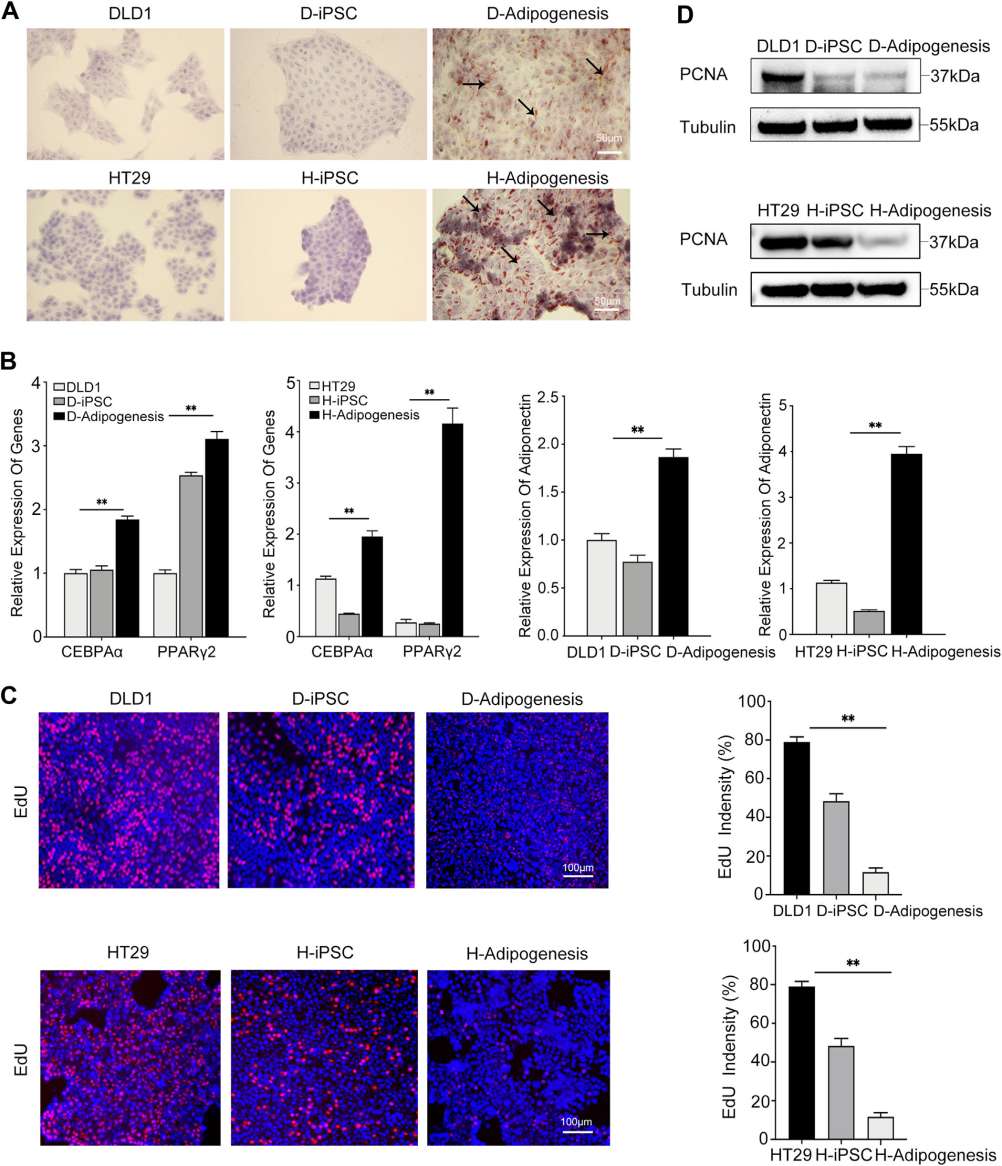
**CRC-iPSCs can effectively differentiate into adipocyte-like cells.***A*, oil-red O staining indicated that the differentiation-induced cells formed rich lipid droplets. Scale bars, 50 μm. *B*, the mRNA levels of adipocyte-specific regulators PPARγ2, C/EBPα, and adipocyte-specific adipocytokine adiponectin were determined by qPCR. (∗*p* < 0.05, ∗∗*p* < 0.01). Data are means ± SD from three technical replicates in one independent experiment and representative of three independent experiments. *C*, differentiated fat cells and controls were allowed to incorporate EdU for 72 h to label proliferating cells and are represented by image panels of EdU (*red*) and DAPI (*blue*) staining. The EdU signal intensity was quantified using ImageJ and is displayed in the right charts. Mean ± SEM (n = 3); (∗*p* < 0.05, ∗∗*p* < 0.01). Scale bars, 100 μm. Images are representative of three independent experiments. *D*, the Western blot results showed that PCNA was expressed at low levels in adipocyte-like cells. Tubulin was used as an endogenous control. Blots are representative of three independent experiments.

We subsequently examined whether the terminal differentiation potential of iPSCs was limited to the neuronal lineage or whether iPSCs had a broader differentiation potential, such as into adipocytes. We conducted a two-step adipogenic differentiation procedure for D-iPSCs and H-iPSCs, involving mesoderm induction. The differentiated adipocytes derived from D-iPSCs and H-iPSCs were named D- and H-adipocytes, respectively. The adipocyte-like cells showed rich LD structures by Oil Red O staining, but these structures were not detected in parental cells or iPSCs (Fig. 4*A*).

Notably, C/EBPα and PPARγ2, two major adipogenic transcription factors, were highly expressed in D- and H-adipocytes. Tissue-specific adiponectin detection further supported functional differentiation into adipocytes (Fig. 4*B*). In addition, differentiated adipocytes, such as differentiated neuron cells, showed weaker proliferative characteristics than iPSCs and their parental cell lines (Fig. 4, *C* and *D*). Together, these data suggest that CRC cells undergoing reprogramming acquire the potential to differentiate into adipocytes and that this differentiation is accompanied by a marked, reduction in cell proliferation.

### Successful differentiation of iPSCs into cardiomyocyte-like cells

**Figure 5:**
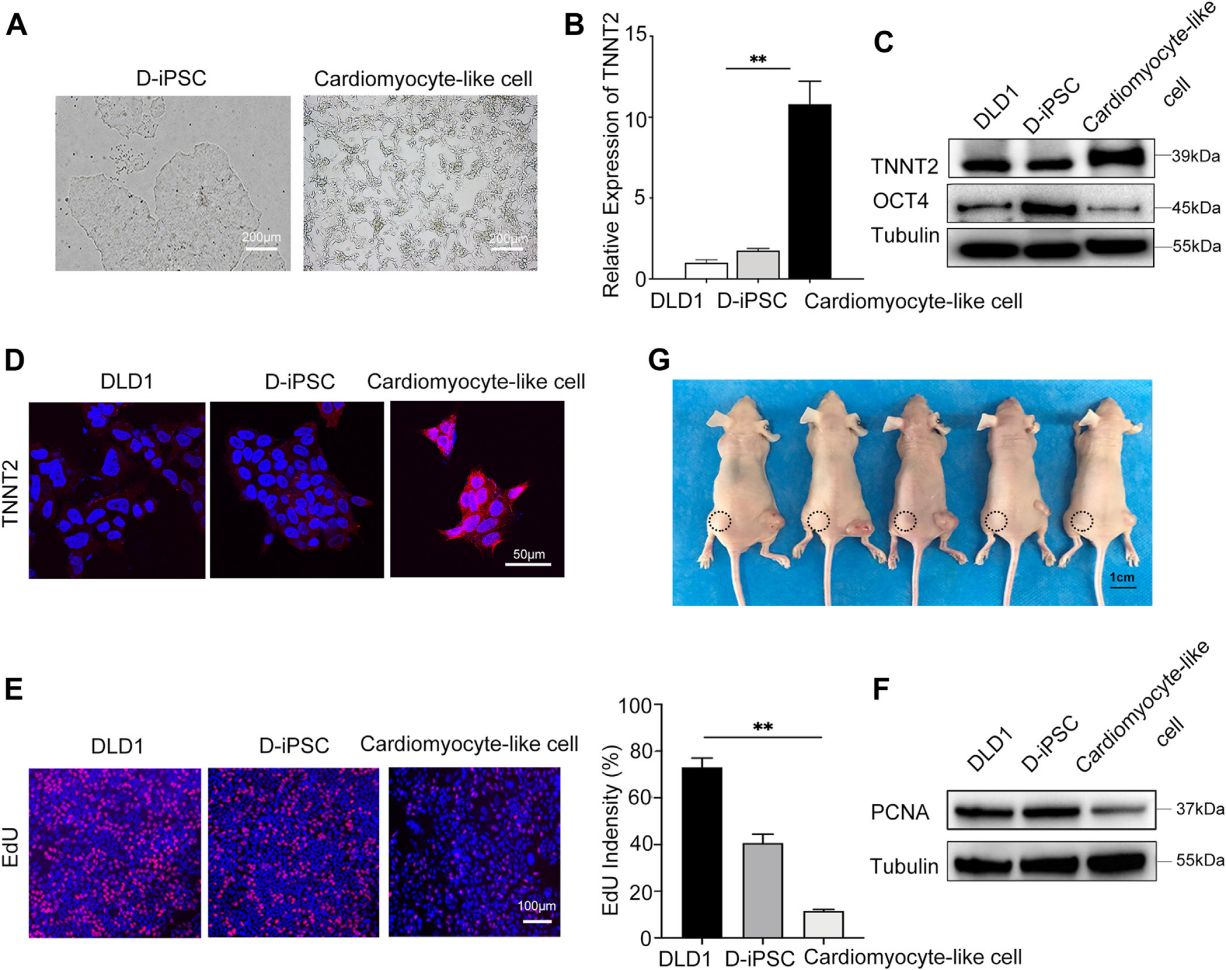
**D-iPSCs terminally differentiated into cardiomyocyte-like cells and obtained nontumorigenicity *in vivo***. *A*, D-iPSCs induced differentiation into cardiomyocytes with marked morphological changes after 8 days. Scale bars, 200 μm. *B*, qPCR results showed significantly higher expression of cardiac troponin T (TNNT2) in cardiomyocyte-like cells than in control cells. (∗*p*< 0.05, ∗∗*p* < 0.01). Data are means ± SD from three technical replicates in one independent experiment and representative of three independent experiments. *C*, Western blot results demonstrated increased expression of TNNT2 and decreased expression of OCT4 in the differentiated cardiomyocytes in comparison with the parental DLD1 cells and D-iPSCs, which served as Controls. Tubulin served as an endogenous control. Blots are representative of three independent experiments. *D*, immunofluorescence showed that the induced cardiomyocytes expressed the marker TNNT2. Scale bars, 50 μm. Images are representative of three independent experiments. *E*, to label proliferating cells, control and cardiomyocyte-like cells were allowed to incorporate EdU for 72 h. Representative images of EdU (*red*) and DAPI (*blue*) staining are shown in the order of DLD1, D-iPSCs, and cardiomyocyte-like cells, and a chart of intensity quantified using ImageJ is then shown. Mean ± SEM (n = 3); (∗*p* < 0.05, ∗∗*p*< 0.01). Scale bars, 100 μm. Images are representative of three independent experiments. *F*, the Western blot results showed that PCNA was expressed at low levels in cardiomyocyte-like cells. Tubulin served as an endogenous control. Blots are representative of three independent experiments. *G*, after 5 weeks, the xenograft tumors were barely visible on the dorsal flanks of nude mice injected on the left side with the differentiated cardiomyocytes (*dot-lined*), and an obvious mass was observed on the opposite side, which was injected with DLD1 cells.

We attempted to differentiate the iPSCs of CRC cell lines into cardiomyocytes. This process took only 15 days, much shorter than that of neuronal cells. Specifically, the treated iPSCs showed significant morphological changes on the eighth day that clearly distinguished them from their parental control cells (Fig. 5*A*). On day 15, qPCR and western blotting were conducted to detect the expression of cardiac troponin T (TNNT2), which is a characteristic biomarker of cardiomyocyte-like cells and is mainly localized in the myocardium (28). TNNT2 was much more highly expressed in D-iPSC differentiated cardiomyocytes than in DLD1 cells and D-iPSCs (Fig. 5, *B* and *C*). Immunofluorescence also showed that the reprogrammed iPSCs successfully differentiated into cardiomyocytes (Fig. 5*D*). Similarly, the 5-ethylene-2′-deoxyuridine incorporation assay and the extremely low PCNA expression demonstrated an almost complete loss of cell proliferation ability after differentiation *in vitro* (Fig. 5, *E* and *F*). To investigate whether the cardiomyocytes produced from reprogrammed cancer cells had attenuated tumor growth *in vivo* after differentiation, we injected the cardiomyocyte-like cells and their parental control cells simultaneously into the right and left flanks of nude mice. Seven days after injection, D-iPSCs clearly developed visible tumor masses, whereas no tumors were detected at the injection sites of cardiomyocyte-like cells. We also did not observe any tumor formation in the mice injected with cardiomyocyte-like cells for up to 5 weeks (Fig. 5*G*). These results suggest that differentiated cardiomyocyte-like cells lose their parental tumorigenicity *in vivo*.

### Epigenetic changes occurred after reprogramming and transdifferentiation with reduced tumorigenicity

**Figure 6:**
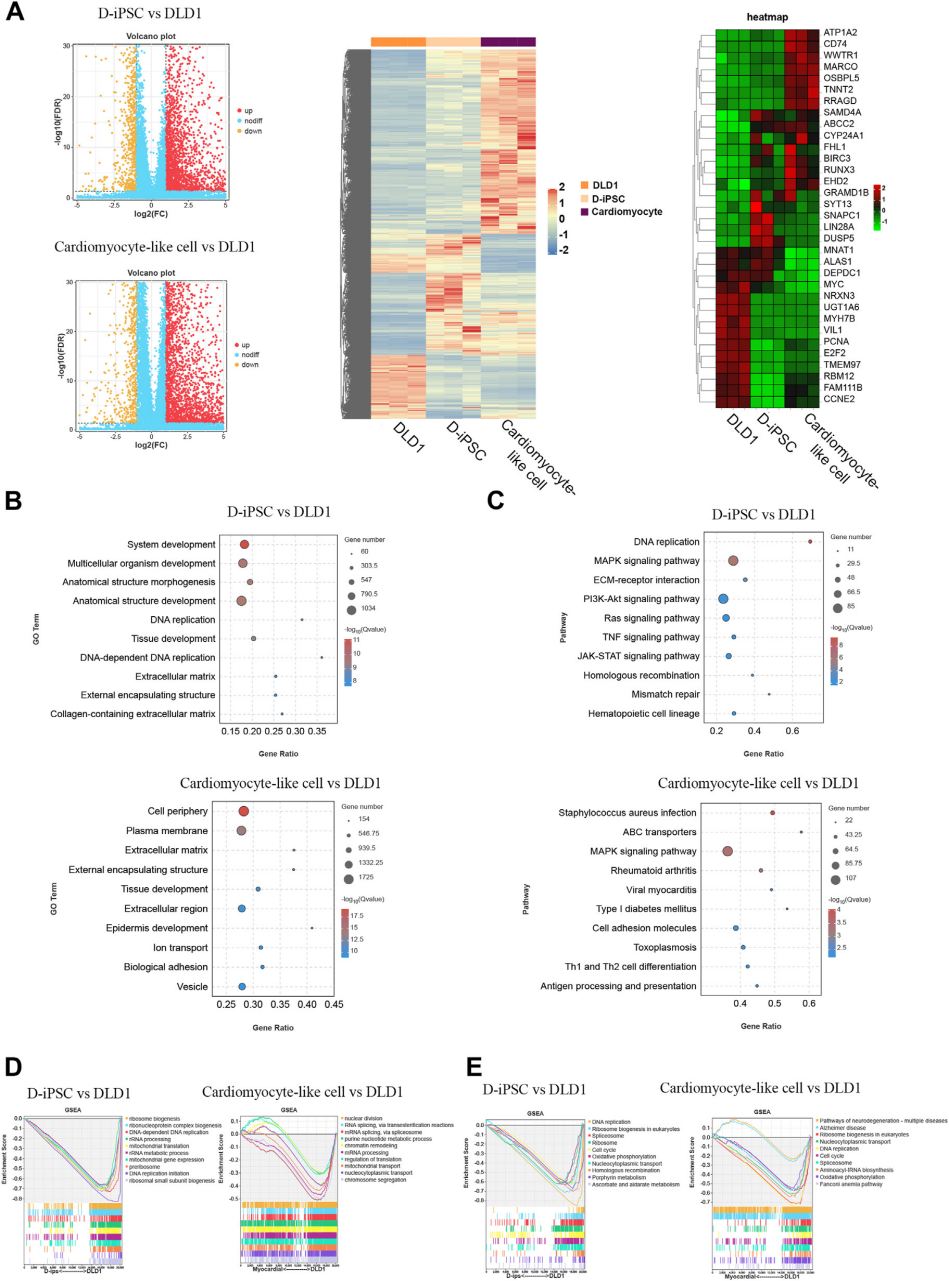
**RNA-Seq analysis of DLD1, D-iPSCs, and cardiomyocyte-like cells.***A*, volcano plot and heatmaps of RNA-Seq results showed transcriptional changes in DLD1 cells after reprogramming and differentiation. (fold change > 2 and false discovery rate (FDR) <0.01). Data from three independent experiments. *B*, GO analysis showed the pathway enrichment of D-iPSCs vs DLD1 cells and cardiomyocyte-like cells vs DLD1. (fold change > 2 and false discovery rate (FDR) <0.01). *C*, KEGG analysis showed the pathway enrichment of D-iPSCs vs DLD1 and cardiomyocyte-like cells vs DLD1. (fold change > 2 and false discovery rate (FDR) <0.01). *D*, GSEA map showed the gene enrichment in the main GO pathways in D-iPSCs vs DLD1 and cardiomyocyte-like cells vs DLD1. (fold change > 2 and false discovery rate (FDR) <0.01). *E*, The GSEA map shows the gene enrichment of the main KEGG pathways in D-iPSCs vs DLD1 and cardiomyocyte-like cells vs DLD1. (fold change > 2 and false discovery rate (FDR) <0.01).

We sought to explore the mechanisms of reduced or even lost tumorigenicity by examining differentially expressed genes after reprogramming and transdifferentiation. Using RNA sequencing (RNA-seq) among DLD1, D-iPSC, and the cardiomyocyte-like cells, we found that 2174 transcripts were upregulated and 729 transcripts were downregulated in D-iPSC compared with DLD1. Meanwhile, 3569 genes were up-regulated and 1149 genes were down-regulated in cardiomyocyte-like cells compared with DLD1 (fold change > 2 and false discovery rate (FDR) <0.01, Fig. 6*A* left). All the gene expression profiles were presented as heatmaps, and the comparison of DLD1, D-iPSC, and the cardiomyocyte-like cells showed great differences in gene expression, indicating great epigenetic changes occur in the process of cancer cell reprogramming and transdifferentiation. In addition, the top 20 differentially expressed genes among the three groups were also presented (Fig. 6*A* right). Among them, DEPDC1 was predicted to enable GTPase activator activity and plays a pivotal role in the regulation of proper mitotic progression (29, 30). ALAS1 is a rate-limiting enzyme in heme biosynthesis (31), and MNAT1 (menage a trois 1, MAT1) is a cyclin-dependent kinase-activated kinase (CAK) complex (32). All these 3 molecules are highly expressed in D-iPSC and the cardiomyocyte-like cells compared with the parental DLD1 cells, which are also highly expressed in diverse cancers and are involved in cancer molecular pathogenesis. Interestingly, TnnT2, the representative cardiac structural genes cardiac troponin T, was specifically expressed in cardiomyocyte-like cells, while Lin28A, a marker of pluripotency (33), was specifically expressed in D-iPSC. The proliferation marker PCNA also showed a trend of low expression after reprogramming and transdifferentiation.

The analysis of the GO enrichment pathway showed that the reprogramming of DLD1 was related to differentiation and tissue development signaling pathways (Fig. 6*B*), this is consistent with the differentiation potential of iPSC. Pathways classified with KEGG are concentrated, mainly on cellular functions such as DNA replication (Fig. 6*C*). The results of GSEA gene enrichment analysis showed that the DNA replication, ribosome genesis, and cell cycle genes were significantly downregulated in D-iPSC and cardiomyocyte-like cells compared with the parental DLD (Fig. 6, *D* and *E*).

## Discussion

In terms of CRC treatment, researchers have recently proposed that a “cancer state” can be reversed into a normal state through epigenetic reprogramming. A fact of cancer cell reprogramming is that these cells can be reversed into a pluripotent state. The remodeling of oncogenes and tumor suppressor genes may lead to a reversal of the “cancer state” (34, 35).

Although somatic cell reprogramming has been extensively studied and direct transdifferentiation of tumor cells into new lineages has been investigated (36, 37, 38), few studies have investigated the reprogramming plus induced lineage-specific differentiation of CRC cells. In the present study, the srRNA vector reproRNA-OKSGM was applied to transfect CRC cell lines efficiently. The reprogrammed D- and H-iPSCs formed tightly packed colonies, and endogenous pluripotency biomarkers were successfully induced. It is not detectable that the reprogramming procedures change the karyotypic status in the iPSC cells, even when SNV and CNV were not performed. These results show that srRNA ReproRNA-OKSGM was successfully applied for the reprogramming of CRC cells *in vitro*.

Transformation of normal cells to malignant cells has been the focus of cancer studies, but it is still unknown whether cancer cells may be effectively transformed back to their normal state (39). An increasing number of constitutive carriers of cancer genetic predispositions, such as BRCA1/2, TP53, Lynch syndrome mutations, and Noonan syndrome mutations, have been found due to the advances in clinical genomic tests with high-throughput sequencing techniques. Mutations of oncogenes may be inherited and arise anytime throughout life without any tumor development. An oncogenic mutation is usually involved in certain tissues other than others. Theoretically, the reprogramming/dedifferentiation of cancers followed by their differentiation to other tissues, especially a terminal differentiation tissue or to rarely tumorigenic tissues is feasible. To experimentally achieve this, we investigated the malignant phenotype of iPSCs reprogrammed from cancer cells. Our study showed that OKSGM expression not only converted CRC cells into iPSCs but also induced cell cycle slowdown and reduced the cell growth rate. Further *in vivo* experiments also exhibited decreasing tumorigenesis. The expression of a single transcription factor, such as OCT4 or SOX2, may make colorectal cancer cells more aggressive (40, 41, 42, 43). In our study, the coexpression of multiple pluripotency factors not only led to a pluripotent status but also reduced tumorigenesis of the CRC cells.

Recent studies found that the transdifferentiation of pancreatic cancer cells into normal epithelial cells (44), or the transdifferentiation of breast cancer cells into adipocytes (45) could effectively reduce their malignancy. The established AML-iPSC model showed multiple paths to differentiate, in which the hematopoietic lineage differentiation from the reprogrammed human myeloid iPSCs regained tumorigenicity; nevertheless, the nonhematopoietic lineages, such as cardiomyocytes derived from the same AML-iPSCs lost their BCR-ABL dependent cancerous features (46, 47). To achieve complement differentiation of CRC-iPSC cells (48), we tried to induce the pluripotent cells into cardiomyocytes, neurons, and adipocytes because these types of cells had the potential to terminally differentiate, by which the cells might lose their ability to proliferate autonomously. We successfully induced CRC-iPSCs into cardiomyocytes, neurons, and adipocytes *in vitro*. The expression of the pluripotent gene OCT4 greatly decreased as iPSCs differentiated into cardiomyocytes, neurons, and adipocytes. The positive cell lineage markers indicated the successful differentiation of the iPSCs. Oncogenic mutations (*KRAS* and *BRAF* mutations) did not block reprogramming dedifferentiation and later on transdifferentiation into cardiomyocyte, neuron, and adipocyte lineages *in vitro*. Moreover, *in vitro* proliferation assays showed that the proliferative rate was substantially weakened or lost after transdifferentiation. To verify the efficacy of this treatment, we further conducted tumorigenic experiments with the differentiated cardiomyocytes in nude mice and found that those cells lost their tumorigenic potency *in vivo*. All these data suggested that the transdifferentiation of CRC cells into cardiomyocyte cells may be an optimal strategy, not only because of their loss of tumorigenicity *in vivo* but also the simplicity of the induction process, and the nature of autonomous terminal differentiation.

Further RNA-Seq results revealed that the mechanisms of decreasing or even loss of tumorigenicity were probably attributed to the control and regulation of oncogenic expression in tumorigenic and differentiating pathways. DNA replication and ribosome production are related to cell proliferation in tumor cells (49). Our data displayed the reduction of tumorigenicity may be caused by decreasing ribosome production, DNA replication, and cell cycle gene expression. The underlying mechanisms probably lie in the epigenetic changes through powerful reprogramming and multi-lineage differentiation induction.

However, there are still limitations to our research. First, we noticed that the reprogrammable nature of CRC cells is not suitable to all CRC lines, as we attempted to reprogram other two CRC cell lines but failed. We suppose that this reprograming heterogeneity is due to different genomic mutation backgrounds and maybe even epigenetic alterations in different CRC cell lines. Second, the application of our research to clinical cancer treatment still faces significant challenges. Last, due to the carcinogenic risk introduced by expressing reprogramming transcription factors, it would be interesting to study more combos of transcription factors and chemical drugs. Together, our study expands the understanding of cancer cell reprogramming, provides a proof of concept, and encourages a potential path for CRC-differentiation therapy.

## Experimental procedures

### CRC cells and culture medium

The human CRC cell lines DLD1 and HT29 were stored in the State Key Laboratory of Cancer Biology, STR identification correct. They were cultured in Dulbecco’s modified Eagle’s medium (DMEM) (Gibco) supplemented with 10% fetal bovine serum (FBS, Gibco) and 1% penicillin‒streptomycin (Gibco) and incubated at 37 °C in 5% CO_2_.

### Generation of iPSCs from CRC cell lines

To reprogram the human CRC cell lines, DLD1 and HT29 (5 × 10^4^ cells/cm^2^) cells were plated on Matrigel (Corning)-coated 6-well plates in growth medium. The growth medium consisted of Advanced DMEM (Gibco), 10% FBS, 1% L-glutamine (Gibco), and 175 ng/ml Recombinant B18R Protein (STEMCELL Technologies). Transfection was started after the cell growth reached 40%-50% confluence.

The ReproRNA cocktail, which included OKSGM (OCT3/4, SOX2, GLIS1, KLF4 and c-MYC), transfection supplement, and transfection reagent (STEMCELL Technologies), was then used for cell transfection. After 24 h, the cell medium was replaced with growth medium. After 48 h, the untransfected cells were removed by adding a concentration of 0.8 mg/ml of puromycin. The cells that survived 3 to 5 days after selection were considered pre-iPSC colonies. The pre-iPSC colonies were all passaged and cultured in ReproTeSR medium (STEMCELL Technologies) with recombinant B18R protein for 2 weeks to expand iPSC colonies. By day 30, iPSC colonies were picked and passaged, the colonies with typical iPSC morphological characteristics were randomly collected and cultured in 3 to 5 different wells and then cultured in mTeSR 1 medium (STEMCELL Technologies). It was noted that the functions of all colonies of iPSC were consistent.

### Culture and passage of CRC cell line iPSCs

Undifferentiated iPSCs derived from reprogramming were plated on Matrigel-coated 6-well plates in mTeSR 1 medium (STEMCELL Technologies, Canada). The medium was changed daily during the culture process. Immediately the differentiation of iPSCs was observed, and the cells were removed. The iPSCs were then passed every 3 to 7 days. Once the cell confluence reached 80%, the cells were dissociated using a gentle cell dissociation reagent (GCDR) (STEMCELL Technologies) for 5 to 10 min. The cells were gently resuspended in mTeSR 1 medium (with ROCK inhibitor added only on the first day of passage at a final concentration of 10 μM/ml) and transferred to matrigel-coated tissue culture plates and cultured in 5% CO2 at 37 °C.

### iPSC differentiation into cardiomyocytes

iPSCs (8 × 105 cells/cm2) were inoculated on 12-well substrate-coated plates to induce differentiation. Before differentiation therapy, iPSC confluence reached 95%. On day 0, the matrix glue was diluted at 1:10 using precooled STEMdiff cardiomyocyte differentiation medium A (STEMCELL Technologies). After 2 days of culture using this medium, another medium was used following the instructions on the STEMdiff cardiomyocyte Differentiation Kit. Cardiomyocytes differentiated from iPSCs can be used for follow-up experiments and validation, and their characteristics can be maintained for 1 month.

### iPSC differentiation into neurons

We used the method of monolayer culture differentiation to induce iPSCs to differentiate into neural progenitor cells and then into neurons. The culture was first coated with poly-l-ornithine (PLO)/laminin (Sigma‒Aldrich). Passaging was conducted using mTeSR 1 (STEMCELL Technologies) medium supplemented with the ROCK inhibitor Y-27632 (STEMCELL Technologies). After 24 h, the Stemdiff neural induction medium supplemented with SMADi (STEMCELL Technologies) was used for 12 consecutive days, and we changed the medium every day for differentiation culture. On day 6, accutase (STEMCELL Technologies) was used to dissociate the cells. On days 12 to 18, Stemdiff forebrain neuron nutrition medium (STEMCELL Technologies) was used for the next step of culture to induce differentiation.

The neurons that differentiated between 18 and 21 days were inoculated into one of the 6-well plates of Poly-l-ornithine (PLO)/laminin plates at a density of 5 × 10^5^ cells/well. During this period, we changed the Stemdiff neural induction medium + SMADi (STEMCELL Technologies) every day. From days 21 to 28, the cells were incubated in Stemdiff forebrain neuron differentiation medium. Neuron maturation was then continued in Stemdiff forebrain neuron maturation medium (STEMCELL Technologies) for a minimum of 8 days.

### iPSC differentiation into adipocytes

D-iPSCs and H-iPSCs were differentiated into adipocytes. Mesoderm differentiation was induced on day 0 in the mesoderm induction medium (STEMCELL Technologies). On day 4, adipocyte differentiation was induced in an adipogenic differentiation medium (STEMCELL Technologies) with 1% L-glutamine. After 2 to 3 weeks, adipocytes had formed, and the presence of lipid droplets (LDs) was identified by Oil Red O (ORO) staining.

### Immunofluorescence

CRC cells (2 × 10^5^ cells/well) were seeded on chamber slides (Millicell EZ Slide, 4-well Glass), whereas neuron- and cardiomyocyte-like differentiated cells were seeded on Matrigel-coated chamber slides and cultured for 48 h. The samples were fixed using 4% paraformaldehyde (PFA) (Sigma‒Aldrich) for 15 min at room temperature. The cell membranes were then permeated with 0.5% Triton X-100 (Thermo Fisher Scientific) for 10∼15 min. The cells were then incubated in 0.5% BSA for 2 h at room temperature. The samples were subsequently incubated with the following primary antibodies overnight at 4 °C in blocking solution at the indicated dilutions: anti-NANOG antibody (dilution 1:200, #4903S, Cell Signaling Technology), anti-OCT4 (dilution 1:200, #Ab19857, Abcam), anti-SOX2 (dilution 1:200, #3579, Cell Signaling Technology), anti-MAP2 (dilution 1:200, #Ab281588, Abcam), and anti-Cardiac Troponin T (dilution 1:200, #Ab209813, Abcam), and anti-Nestin (dilution 1:200, #14-5843-80, Thermo Fisher Scientific). The samples were washed twice with PBS and incubated with the secondary antibodies Alexa Fluor 488 Donkey Anti-Mouse IgG and Alexa Fluor 594 Donkey Anti-Rabbit IgG (Jackson ImmunoResearch). After 2 h at 4 °C, the nucleus was counterstained with 0.1 μg/ml DAPI (Thermo Fisher Scientific) and washed three times with PBS. The labeled cells were observed under a confocal microscope (FV3000, Olympus).

### Animal experiments

In order to analyze the growth and differentiation potential of CRC cells after reprogramming *in vivo*, 7 × 10^6^ undifferentiated iPSCs (D-iPSCs and H-iPSCs) cultured to the eighth generation and control cells of parental CRC were resuspended in DPBS, and healthy nude mice aged 6 to 8 weeks were selected for subcutaneous injection of the two types of cells. There were 5 nude mice in each group, and the growth state and tumor formation of nude mice were observed continuously. After tumor formation, the tumor volume was measured every 3 days, and the nude mice were sacrificed at 4 weeks. After the tumors were collected and photographed, the collected tumors were immobilized in 10% neutral buffer formalin for use in subsequent experiments.

### Statistical analysis

Data are expressed as mean ± standard deviation (SD). Student’s test (two-tailed), Pearson’s correlation coefficient, and one-way analysis of variance were used for data analysis. Differences were considered significant if ∗*p* < 0.05 or ∗∗*p* < 0.01.

## Data availability

The primer data that support the findings of this study are available in the supplementary material file. Further inquiries can be directed to the corresponding author.

## Supporting information

This article contains supporting information.

## Conflict of interest

The authors have no relevant financial or nonfinancial interests to disclose.
