# Supplementary material for: Reprogramming and multi-lineage transdifferentiation attenuate the tumorigenicity of colorectal cancer cells
Source: J Biol Chem. 2023 Dec 10;300(1):105534. doi: 10.1016/j.jbc.2023.105534 (PMC10801221; doi:10.1016/j.jbc.2023.105534)
Supplement: Supplementary material [file mmc1.docx]

**Experimental Procedures**

**Karyotyping analysis**

Karyotyping analysis was performed according to the manufacturer’s instructions for the NGKTM kit (RJY, Beijing). In brief, DLD1 and HT29 cell lines and their induced pluripotent stem cells D-iPSCs and H-iPSCs were cultured for 3 days in plastic cultures. All cell lines were pretreated with 1 mg/ml 9-aminoacridine for 90 min, and mitotic arrest was induced with colchicine (0.5 µg/ml, 20 min). Buffered hypotonic solution (NGK01) was used to help disperse chromosomes for 15 min at 37 °C. After fixation with methanol/acetic acid (3:1), the cell suspensions were dropped onto cold moist slides. NGK02 was used to further dissolve chromosomal bands. The slides were stained in Giemsa solution. The karyotype was described according to the International System for Human Cytogenomic Nomenclature (ISCN 2020).

**Quantitative real-time polymerase chain reaction (qPCR)**

Total RNA extraction was performed using an RNeasy kit (Thermo fisher Scientific, USA) according to the manufacturer’s instructions. The Prime Script RT Reagent Kit (TaKaRa, Japan) was used to reverse transcribe cDNA according to the manufacturer’s advice. Before use, cDNA was diluted 1:5 in nuclease-free H2O. All RT‒qPCR primers were purchased from RiboBio (China). qPCR was performed using TB Green Fast qPCR Mix (TaKaRa, Japan). The CFX96 Real-Time PCR Detection System (Bio-Rad, USA) was used for quantification. The following primers were used in the study:

| TNNT2 | Forward: AGACAGAGCGGAAAAGTGGG |
| --- | --- |
|  | Reverse: TCCTTGGCCTTCTCCCTCAG |
| PPARγ2 | Forward: CCTCGAGGACACCGGAGAG |
|  | Reverse: CACGGAGCTGATCCCAAAGT |
| CEBPα | Forward: AAACAACGCAACGTGGAGA |
|  | Reverse: GCGGTCATTGTCACTGGTC |
| Adiponectin | Forward: GTGAGAAGGGTGAGAAAGGAGAT |
|  | Reverse: CACACTGAATGCTGAGCGGTATA |

**Flow cytometry**

Accutase (STEMCELL Technologies, Canada) was used to harvest CRC cells and CRC-iPSCs. After fixation with 75% cold ethanol overnight, the cells were incubated with RNase-free water for 30 min. Next, propidium iodide was used to stain the cells on ice and in the dark for 30 min. MODFIT software (BD) was used to quantify the cell cycle distribution.

**Cell Counting Kit-8 (CCK-8) assay**

A CCK-8 assay (GLPBIO, Montclair, CA, USA) was used to determine the viability of CRC cells. Cells were seeded at a density of 1 × 10^3^ cells per well and cultured in a 96-well plate. At 0, 1, 2, 3, 4, and 5 days, 10 µl of CCK-8 reagent was added to each well and incubated at 37°C for 2 h. The absorbance of each well was detected at 450 nm by a VARIOSKAN FLASH system (Thermo Fisher Scientific, USA).

**Immunohistochemistry (IHC)**

The sides were heated on a 65°C heating panel for 60 min, deparaffinized, incubated with 3% hydrogen peroxide, and blocked overnight at 4°C by avidin-biotin. After incubation with mouse anti-human Ki-67 primary antibody (dilution 1:150, #9449, Cell Signaling Technology, USA), the sections were incubated with horseradish peroxidase (HRP)-conjugated secondary antibodies (ZSGB, Beijing, China). Afterward, the samples were counterstained with hematoxylin. All samples were analyzed under a Leica DM LS light microscope.

**Western blot analysis**

Protein was extracted from cells or tissue as follows: ice-cold RIPA lysis (Beyotime, China) was supplemented with phosphatase and protease inhibitors and used to cover cells or tissues on ice for 10 min. The protein concentration was detected by the BCA Protein Assay Kit (Thermo fisher Scientific, USA). SDS‒PAGE was used to separate denatured proteins from cells or tissues. Proteins were transferred to nitrocellulose filter membranes (Millipore, USA). Next, the membrane was incubated with 10% skim milk for 1 h at room temperature and then incubated with the primary and secondary antibodies. The protein band was exposed and detected by a Bio-Rad Imaging System (Bio-Rad, USA). The antibodies used were as follows: Anti-NANOG antibody (dilution 1:1000, #4903S, Cell Signaling Technology, USA), Anti-OCT4 (dilution 1:1000, #Ab19857, Abcam, UK), Anti-SOX2 (dilution 1:1000, #3579, Cell Signaling Technology, USA), Anti-MAP2 (dilution 1:1000, #Ab281588, Abcam, UK), and Anti-Cardiac Troponin T (dilution 1:1000, #Ab209813, Abcam, UK), Anti-PCNA antibody (dilution 1:1000, #13110T, Cell Signaling Technology, USA).

**EdU incorporation for detection of proliferating cells**

After adipocyte induction was complete, 5000 cells per well were seeded in a 96-well plate. Cells were incubated with EdU (Apollo 567) (RiboBio, China) at 10 μM for 2 h. Cells were fixed with 4% PFA for 20 min and washed with PBS, and cells permeated with 0.5% Triton X-100 in PBS were washed with PBS. DNA immunostaining was performed according to the manufacturer's protocol. Images were acquired with a fluorescent Leica DMI 4000 microscope. The Hoechst staining signal coincided with the DAPI staining signal as a positive signal. For each replicate, at least three were quantified using the intensity quantization function in ImageJ software.

**Oil Red O staining**

An Oil Red O staining kit (Solarbio Life Science, Beijing) was used to identify adipocyte production. The manufacturer's protocol was followed. Cells were fixed with PFA, washed with 60% isopropanol for 20–30 s, stained with oil red O staining solution for 20 min, and stained with hematoxylin staining solution for nuclei. After the cells were washed with 60% isopropanol until the intercellular space was clear, they were washed with distilled water for 1 min and observed under a microscope.
